# Advances in Nanomaterials-Based Electrochemical Biosensors for Foodborne Pathogen Detection

**DOI:** 10.3390/nano11102700

**Published:** 2021-10-13

**Authors:** Ivan Bobrinetskiy, Marko Radovic, Francesco Rizzotto, Priya Vizzini, Stefan Jaric, Zoran Pavlovic, Vasa Radonic, Maria Vesna Nikolic, Jasmina Vidic

**Affiliations:** 1 BioSense Institute, University of Novi Sad, 21102 Novi Sad, Serbia; bobrinet@biosense.rs (I.B.); marrad@biosense.rs (M.R.); sjaric@biosense.rs (S.J.); zoran.pavlovic@biosense.rs (Z.P.); vasarad@biosense.rs (V.R.); 2 Micalis Institute, INRAE, AgroParisTech, Université Paris-Saclay, 78350 Jouy-en-Josas, France; francesco.rizzotto@inrae.fr; 3 Department of Agriculture Food, Environmental and Animal Sciences, University of Udine, 33100 Udine, Italy; vizzini.priya@spes.uniud.it; 4 Institute for Multidisciplinary Research, University of Belgrade, 11030 Belgrade, Serbia

**Keywords:** electrochemical biosensing, nanomaterials, graphene, carbon nanomaterials, gold nanoparticles, metal oxides, quantum dots, 2D nanomaterials, foodborne pathogen

## Abstract

Electrochemical biosensors utilizing nanomaterials have received widespread attention in pathogen detection and monitoring. Here, the potential of different nanomaterials and electrochemical technologies is reviewed for the development of novel diagnostic devices for the detection of foodborne pathogens and their biomarkers. The overview covers basic electrochemical methods and means for electrode functionalization, utilization of nanomaterials that include quantum dots, gold, silver and magnetic nanoparticles, carbon nanomaterials (carbon and graphene quantum dots, carbon nanotubes, graphene and reduced graphene oxide, graphene nanoplatelets, laser-induced graphene), metal oxides (nanoparticles, 2D and 3D nanostructures) and other 2D nanomaterials. Moreover, the current and future landscape of synergic effects of nanocomposites combining different nanomaterials is provided to illustrate how the limitations of traditional technologies can be overcome to design rapid, ultrasensitive, specific and affordable biosensors.

## 1. Introduction

Pathogen diagnostics are currently critical for applications in healthcare, food safety analysis and environmental monitoring. Foodborne and waterborne pathogens (i.e., bacteria, fungi, viruses and some parasites) cause infections in humans via contaminated food or water. The high incidence of infection caused by foodborne pathogens indicates that the prevention, surveillance and management of foodborne diseases need to be strengthened [1,2,3].

The traditional technologies to detect pathogens in food and water are constrained by delayed analysis times, expensive and laborious sample preparation steps and the need for highly trained personnel. The major conventional detection methods can be classified as counting methods, immune-assays and polymerase chain reaction (PCR)-based methods. Counting bacterial colonies on microbiological culture plates is inherently a complex, time-consuming and error-prone method. The detection time takes 3 to 9 days while up to 2 weeks are needed for confirmation of positive results. The confirmation includes observation of the bacterial colony color and morphology together with biochemical tests in a specific medium that is performed after pathogen isolation. Alternatively, immunoassays, such as the enzyme-linked immunosorbent assay (ELISA), lateral flow and dot blot immunoassay, enable detection of pathogen antigens [4,5,6,7,8]. They can use monoclonal or polyclonal antibodies that specifically bind to the targeted pathogen and can be applied for testing large-scale samples and for the on-site detection of pathogens. However, they usually show low sensitivity and thus have to be confirmed by an additional test. In contrast, PCR-based methods allow rapid and highly specific pathogen diagnosis. In spite of these advantages, PCR-based methods have some limitations. For instance, they can produce false negative results due to a DNA polymerase inhibition by food matrix molecules and ions, which may completely block amplification of target DNA, or false positive results due to the cross-amplification of PCR-generated fragments of non-target DNA.

Biosensors provide a promising tool for such applications due to their portability and simplicity of utilization. The most used types of sensors are by construction optical (plasmonic, UV-Vis/Infrared spectroscopy, Raman, attenuated total reflection), electrochemical, electromagnetic, mechanical, airflow and acoustic. The principal issue in all these technologies is to enable sensitive and selective detection of pathogens in complex food samples that contain low analyte concentrations. Nonspecific adsorption of biomolecules presented in the sample (originated from either the matrix or microorganisms that constitute normal sample microflora) at the biosensor surface can drastically obstruct detection performance, diminish the signal intensity and specificity of the biosensor and increase background “noise”.

In the last years, innovative and portable biosensors have emerged as they overcome limitations of traditional and molecular detection technologies and even other biosensors concerning the quantitative detection and screening of pathogens in clinical, environmental and food analysis [9,10]. Among different biosensors, electrochemical platforms are the most popular because they are highly specific towards the analyte and can be adapted for multiplex analysis providing high analytical accuracy even in complex food matrices of various composition, densities and pH. Electrochemical detection of a pathogen exploits a working electrode modified with specific recognition elements (such as antibody, aptamer, DNA probe) ensuring the selectivity, sensitivity and specificity of the measurements. Various strategies and concepts have been developed to prevent nonspecific binding to the electrode surface in biosensors. The concept of such strategies primarily relies on the fabrication method, sample composition, electrochemical technique and performance of each detection principle. Recent literature highlights that different nanomaterials are incorporated into electrochemical biosensors as enhancers, labeling factors or immobilizer supports to enable the overall feasibility of the platform for diagnostic/detection applications.

In this review, we present some basic principles of the electrochemical methods used in biosensors and the state-of-the-art nanomaterial-based electrochemical biosensors for foodborne pathogen (virus, bacteria and bacterial toxin) detection.

## 2. Electrochemical Methods and Electrode Functionalization

**Figure 1:**
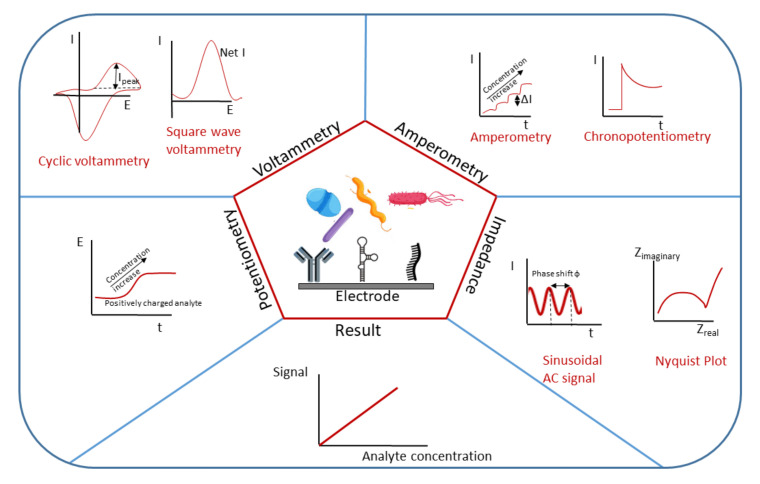
Electrochemical biosensors utilizing different methods (potentiometry, voltammetry, amperometry and electrochemical impedance) for analyte detection and concentration evaluation.

Electrochemical biosensors transduce biochemical events into electrical signals (current, potential, impedance or resistance). They can be divided into biocatalytic using enzymes as recognition elements and affinity (biocomplexing) biosensors using selective and strong binding biomolecules. Affinity biosensors can be further divided into immunosensors based on antibodies or nanobodies, aptasensors based on DNA or RNA aptamers and genosensors based on single strand DNA (ssDNA). Besides, some electrochemical biosensors for pathogen detection use peptides, phages, microRNA, antibiotics or molecularly imprinted polymers (MIPs) as recognition elements [10]. Various electrochemical techniques with different signal mechanisms exist, as illustrated in Figure 1. Their applicability and efficiency depend on the target properties and design of the sensor platform. Voltammetry is performed under controlled potentials when the measured current reflects electron transfer between the sample and the electrode surface. It is possible to measure current values during the potential sweeping towards and backwards while cycling (cyclic voltammetry). By holding the potential of the electrode constant (amperometry), or holding the current constant (potentiometry), the obtained information in the timescale gives the change in current and potential, respectively. Capacitance, as one of the electric properties of (bio)molecular and biological layers at the surface of the electrochemical electrode, represents important information of the layer charging effect while sensing certain molecules/pathogens. Electrochemical impedance spectroscopy (impedance/resistance of the system) is usually employed for measuring the impedance of the catalytic layer that changes upon target binding to the immobilized recognition element. Electrochemical impedance spectroscopy is frequently used in detecting pathogen microorganisms due to its high sensitivity. Increase in the diameter of electrochemical impedance plotted in a Nyquist diagram shows the increase in system impedance. It is directly proportional to the electron transfer resistance of the system and enables quantitative detection. Potentiostatic systems usually work in a three-electrode format (working, auxiliary and reference electrode) while conductometry and electrochemical impedance spectroscopy are mainly performed in a two-electrode format (working and auxiliary).

An ideal electrochemical sensor should achieve high sensitivity and specificity, a wide dynamic range of detection, measurement reproducibility, rapid response with real-time analysis and multiple uses. It should also be portable, user-friendly and cost-effective with self-calibration and self-cleaning. To enable simultaneous detection of several targets in the same sample (multiplexing), electrochemical biosensors can be combined with microfluidic systems and integrated with microelectronics. The development of screen-printed electrodes is important in making sensors economical and widely commercially available. Furthermore, data post-processing plays a very important role in obtaining credible and accurate detection results. Many of these properties are an issue in detecting analytes in complex matrices. Affinity biosensors, especially, may have difficulty operating in samples such as food matrices due to nonspecific adsorption on the electrode surfaces that compromises the performance of the device.

The electrode material, its design and fabrication may significantly increase the sensor specificity and selectivity. Surface chemistry is used to immobilize recognition elements onto the working electrode and to prevent a background signal [11]. To eliminate the matrix effect, common strategies involve electrode functionalization using specific surface chemistry and additional electrode covering with poly(ethylene glycol) (PEG) or oligo(ethylene glycol) (OEG) layers that effectively passivate the electrode [12]. Usually, immobilization of biomolecules is performed via amine-, carboxyl-, aldehyde- and thiol- conjugation, depending on the chemical reactivity of the electrode material and its modifications. Finally, to enable sensitive electrochemical detection, a redox indicator is added to the sample. Ferrocene is the most commonly used redox indicator [10], but others such as protamine [13], tripropylamine [14] or methylene blue [15] also enable estimation of the target concentration by measuring changes in peak intensity. For instance, target binding on the electrode surface may decrease peak intensities of the redox indicator due to the higher electron transfer resistance of the electrode system while increasing the concentration of the captured target. To simplify biosensor utilization and to increase signal intensities, the redox marker can be immobilized onto the electrode surface as shown for influenza A virus detection using conducting copolypyrrole integrating ferrocenyl group electrodes [16,17]. Finally, in cases when the analyte can undergo oxido-reduction on the working electrode itself, no additional redox marker is needed [18].

## 3. Nanomaterial-Based Electrochemical Biosensors

Many types of sensing electrochemical devices come up, and some of them represent a scaled-down lab to a single chip (lab on a chip). However, despite the intense development of electrochemical biosensors, their high sensitivity and reproducibility remain challenging [19]. Employing various nanomaterials may improve analytical performances of electrochemical sensors by signal enhancement [9,20]. Association of nanomaterials with the electrode increases surface area which can boost loading capacities and mass transport of reactants, resulting in signal amplification. Moreover, nanomaterials can be carriers of redox probes to provide sensitive detection or can improve dynamics of redox exchanges, which significantly amplifies the read-out [21].

**Figure 2:**
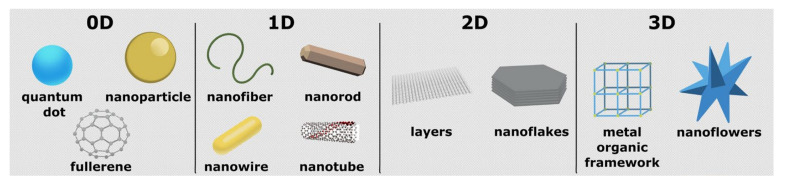
Illustration of some 0–3D nanostructured material morphologies.

Nanomaterials are generally classified as 0D—quantum dots, carbon dots, nanoparticles, 1D—nanotubes, nanowires, nanorods, 2D—nanoplates, nanosheets, nanodisks and 3D—nanoflowers, nanocones, nanoballs [22] (Figure 2). In all 0–3D forms, nanomaterials have been extensively incorporated into electrode construction in electrochemical biosensors applied in the detection of foodborne pathogens [23]. Generally, 0D nanomaterials comprise nanoparticles, usually metal or metal oxide nanoparticles [24,25,26], carbon and quantum dots [27,28] with nanoscale dimensions. Metal nanoparticles, most commonly gold nanoparticles, are often selected for application in electrochemical biosensors for detection of foodborne pathogens due to their high conductivity and biocompatibility and retention of biomolecule activity over time [29,30]. Quantum dots (QDs) have great potential for application in small size electrochemical biosensing devices due to their small compact size and good and stable performance [31]. In terms of material type, quantum dots can be classified as metal QDs, carbon dots (CDs) and graphene quantum dots (GQDs). One-dimensional (1D) nanomaterials applied in electrochemical biosensing generally include carbon nanotubes [32] and metal oxides synthesized in the form of nanowires, nanotubes or nanorods [33]. Two-dimensional (2D) nanomaterials have come into the limelight starting with the discovery of graphene in 2004 [34]. Besides atomic layer thickness, tunable electronic properties, good mechanical strength and chemical activity, they feature a high surface-to-volume ratio making them good candidates for electrochemical biosensing, gas sensing, energy conversion, storage devices and many other biomedical applications [35,36,37,38]. Besides graphene and its derivatives, research has focused on the development of other emerging 2D nanomaterials including boron nitride, graphite carbon nitride, transition metal dichalcogenides, MXenes, black phosphorous, transition metal oxides and also, more recently, heterostructures incorporating at least one 2D nanomaterial [39,40,41]. Metal oxides, when exfoliated into monolayers, can form a 2D oxide nanostructure [40]. Most common 3D nanomaterial structures applied in electrochemical biosensing of foodborne pathogens are generally various metal oxides, often grown in the form of nanoflowers [42] or other 3D structures, and, more recently, carbon allotropes such as laser-induced graphene [43,44]. Especially, nanomaterials with intrinsic conductivity, such as metal oxide, carbon nanomaterials and metal nanoparticles, significantly improve the sensing devices that relied on electrical signal. Moreover, synergic effects can be achieved by combining two or more 0–3D nanomaterials, forming a nanocomposite heterostructure on the same electrode [45,46,47,48].

Different surface modifications involving thiols, amines and silanes are performed to functionalize electrodes carrying nanomaterials in order to attach biorecognition elements to the electrode in a stable way. Proper functionalization is one of the key elements in biosensor development since maintaining the full biological activity upon immobilization allows optimal analytical properties of the biosensor [49]. For instance, DNA probes thiolated at 5′ or 3′, or peptides modified with a cysteine residue at one end, covalently bind to gold film or gold nanoparticles [14], while an antibody can be immobilized covalently via amino links on a gold electrode surface with a previously attached self-assembled thiol layer that was activated with a mixture of 1-ethyl-3-(3-dimethylaminopropyl)carbodiimide (EDC) and N-hyrdoxysuccinimide (NHS) [50]. In addition, non-covalent binding of recognition elements to the electrode via streptavidin–biotin interaction allows for a highly stable biosensor surface [51,52].

### 3.1. Metal Oxide Nanomaterials

Metal oxides are semiconductor materials due to their crystalline ordering, electronic band structure, specific surface and quantum related properties. According to the semi-classical theory [53], the conductivity of a semiconductor can be easily modified/enhanced by changing the concentration or the mobility of free charge carriers. Such features represent an ideal starting point for the design of electrochemical biosensors for pathogen detection. Previous studies have shown that the concentration of free charge carriers in metal oxide materials can be modified by oxygen vacancy concentration, doping, particle size, temperature, humidity, electromagnetic radiation and surface adsorbed species [26,33]. Metal oxide nanomaterials can be synthesized in various morphologies ranging from 0 to 3D, providing an interesting playground for the design of electrochemical biosensors [25]. They are low cost, highly biocompatible, show an antimicrobial effect and have a large catalytic area and electrocatalytic activity [45]. The synthesis procedure has a significant influence on metal oxide nanoparticle morphology and resulting properties [26,33]. Some examples of metal oxide nanostructures include nanoparticles [54,55], nanowires [56], nanocubes [57], nanosheets [58], flower-like structures [59], etc.

**Figure 3:**
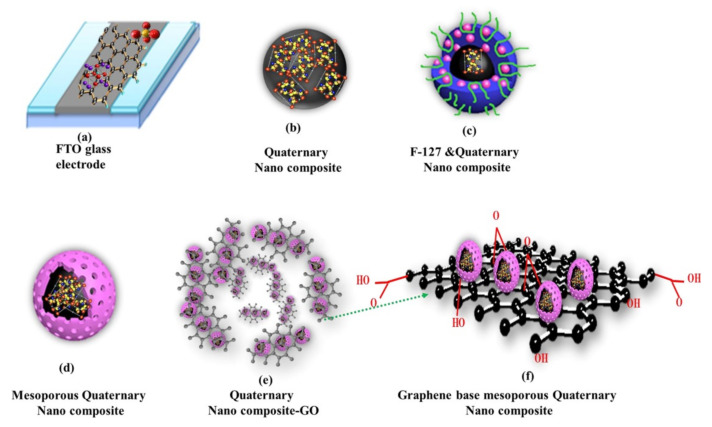
Schematic presentation of the synthesis process of the ZrO_2_-Ag-Graphene Oxide -SiO_2_ nanocomposite. Adapted with permission from [46] Copyright 2020, American Chemical Society.

Recent research has focused on nanocomposite heterostuctures, where different characteristics of each component lead to improved performance and characteristics of electrochemical biosensors for food pathogen detection [46]. Metal oxide 0–3D nanomaterials have great potential to improve the biorecognition performance, where focus is on engineering the microstructure, as shown by Zhai et al., where a 3D networked carbon nanowall/diamond supporting CuO architecture was developed combining microwave plasma chemical vapor deposition of the hybrid carbon nanowall/diamond film on fluorine tin oxide (FTO) glass substrate. It was then used as a template for deposition of Cu nanoparticles by magnetron sputtering followed by growth of CuO nanoparticles by an electrochemical method [45]. Fatema et al. performed a comparative study of two mesoporous nanocomposites, ZrO_2_-Ag-G-SiO_2_ and In_2_O_3_-G-SiO_2_ (Figure 3), for rapid and highly efficient detection of *Escherichia coli* using cyclic voltammetry, achieving detection in the range from 10^1^ CFU/mL to 10^10^ CFU/mL [46].

**Figure 4:**
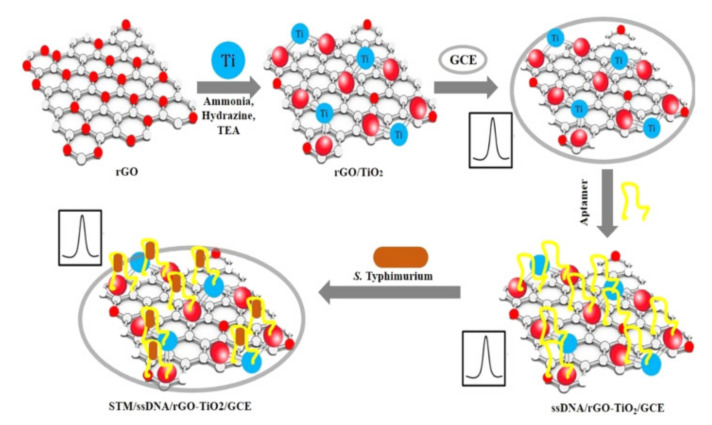
Schematic presentation of the fabrication of rGO-TiO2 electrodes and their employing for electrochemical detection of bacteria. Adapted with permission from [60] Copyright 2019, Elsevier.

Several reports have indicated significant sensing improvements obtained by using metal oxide nanoparticles in electrochemical biosensors. Muniandy et al. [60], developed a reduced graphene oxide–nano TiO_2_ composite for an aptasensor used in the detection of *Salmonella enterica* (Figure 4). The bacterial cells captured by the aptamers incorporated on the electrode surface were a physical obstacle for electron transfer, which decreased the voltammetric signal proportionally to the bacterial concentration. Performance of the sensor was evaluated using cyclic voltammetry and electronic impedance spectroscopy. The developed aptasensor exhibited high sensitivity with a wide detection range (1–10^8^ CFU/mL), low detection limit of 1 CFU/mL, good selectivity for Salmonella strains and acceptable long-term stability. Nadzirah et al. [61] used pure TiO_2_ nanoparticles (NPs) and fabricated interdigitated electrodes for *E. coli* detection. A specific ssDNA probe was immobilized on the electrode surface upon its chemical functionalization with (3-aminopropyl) triethoxysilane (APTES) to provide contact between the organic and inorganic surfaces of a ssDNA probe and TiO_2_ NPs. The obtained genosensor showed high sensitivity since it was able to detect as low as 1.0 × 10^−13^ M of *E. coli* O157:H7 DNA in bacterial lystes, with a high specificity and reproducibility.

Teng et al. [62] showed that ZnO nanorods in an electrochemical immunoassay for detection of *E. coli* led to signal enhancement. They immobilized both the detection antibody (anti-*E. coli* polyclonal antibody) and the redox probe (ferrocene) onto the surface of ZnO nanorods which surfaces were coated with a layer of silica. When coated with silica, the nanorods form core–shell nanorods that can be easily modified with various functional groups. The obtained complex antibody-ZnO-ferrocene was incubated with an *E. coli* contaminated sample and then washed and deposited on to a gold electrode carrying a capturing anti-*E. coli* antibody. The designed immunoassay showed a detection limit of 50 cfu/mL. In another study, Purwidyantri et al. [63] used ZnO nanograss decorated with Au nanospeckles to develop a sensing platform for *Staphylococcus epidermidis*, based on DNA hybridization. Applying the thermal evaporation, Au nanoparticles were deposited on the hydrothermally synthesized ZnO nanograss. The deposition increased by approximately two-fold the effective surface area and diffusion coefficient compared to the non-speckled ZnO nanograss. The fabricated genosensor carrying a DNA probe complementary to the 16S region in the genome of *S. epidermidis* attained a limit of detection (LoD) of 0.506 pM.

**Figure 5:**
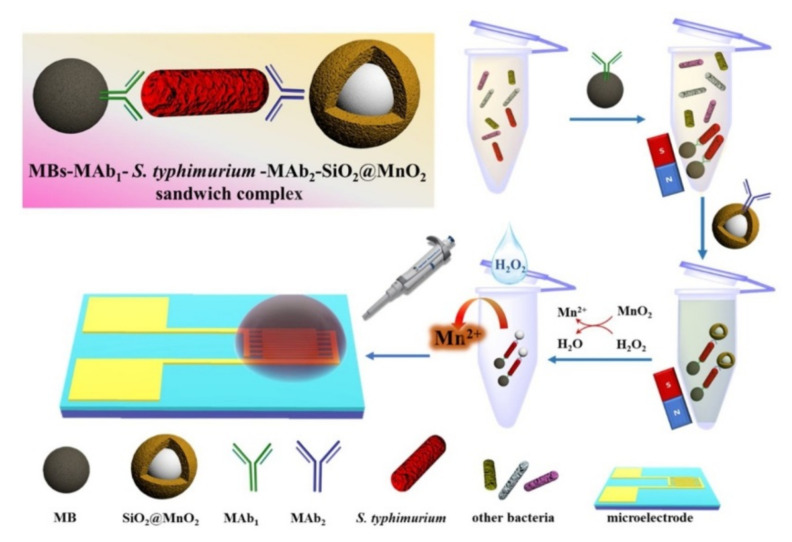
Illustration of the detection of *S. typhimirium* using immunomagnetic separation and impedance monitoring of the release of Mn^2+^ from the SiO_2_@MnO_2_ nanocomposite. Adapted with permission from [65] Copyright 2020, Elsevier.

Earth abundant transition metal oxides are showing great potential for electrochemical applications, including electrochemical biosensors for foodborne pathogens [64]. Thus, rapid detection of *Salmonella typhimurium* was achieved using an SiO_2_@MnO_2_ nanocomposite impedance biosensor developed on interdigitated array microelectrodes combined with immunomagnetic separation [65]. Magnetic beads were used to capture monoclonal antibodies and separate *S. typhimirium* cells rapidly from samples, while detection was achieved by release of Mn^2+^ by H_2_O_2_ monitored as a change in impedance, as shown in Figure 5.

An electrochemical genosensor based on SnO_2_ nanocrystalline quantum dots was developed by Patel et al. [66] for detection of *Vibrio cholerae* using the DNA hybridization principle. The electrode was obtained by electrophoretic deposition of SnO_2_-QDs onto indium-tin oxide coated glass substrate. Subsequently, a DNA probe was attached to SnO_2_ NPs via its phosphate groups. The study showed that SnO_2_ NPs provided not only an effective surface for DNA probe immobilization, but also enhanced electron transport and improved signal read-out. The fabricated sensor detected *V. choleare* complementary DNA sequences with the limit of detection of 31.5 ng/µL and showed high long-term stability.

Bacteria remain the most commonly detected pathogen with metal oxide based electrochemical biosensors. Moreover, current trends are focused on the use of metal oxide photocatalytic properties in inactivation and elimination of bacteria [67,68,69,70]. For instance, a multifunctional electrochemical platform was obtained by combining ZnO, colloidal Ag and vancomycin [71]. AgNPs/3D-ZnO nanorod arrays, functionalized with vancomycin, were immobilized onto the working electrode. The platform detected *S. aureus* with a detection limit of 330 CFU/mL and eliminated bacterial cells with 50% efficiency at low bacterial concentrations of about 10^3^ CFU/mL.

### 3.2. Carbon Nanomaterials

Carbon materials have long been a main companion in electrochemical sensor electrodes, in the form of either glassy carbon or activated carbon due to high chemical inertness and a high specific surface area needed for catalytic material impregnation. The discovery of new carbon allotropes, such as fullerene, carbon nanotubes (CNTs) [72,73] and graphene [74], has triggered active investigation of their application in different types of biosensors. Graphene has a unique two-dimensional honeycomb lattice structure, while the structure of CNTs consists of cylindrical graphene rolled up into a seamless cylinder with a diameter of the order of a nanometer. They provide unprecedentedly high specific surface areas up to 2630 m^2^/g [75] and 1315 m^2^/g [76], respectively, combined with a high electrical conductivity and charge carrier mobility. A number of reviews have discussed the perspectives of graphene and carbon nanomaterial application as materials for electrodes to improve electrochemical sensors [77,78,79], including foodborne pathogen detection [80].

Application of carbon nanomaterials, including graphene-related materials, is fostered by a deeper understanding of their physical and chemical properties as well as scalable production, processing and functionalization methods [81]. Examples of electrochemical biosensors utilizing different carbon nanomaterials are summarized in Table 1. The development of stable solutions of graphene and carbon nanotubes makes them prospective for electrodes integrated with conventional technologies for low-cost disposable electrochemical sensors in point-of-need devices. The graphene and carbon nanotube technology combines well with screen printed technologies for portable electrochemical sensors development [82], as well as paper based devices [83]. In foodborne pathogen detection, graphene-based electrochemical sensors also bring advances as they can operate directly in biological and food matrices. Graphene-based composites with functional nanomaterials and bioreceptors (antibodies, aptamers, DNA probes, etc.) provide low LoD down to pico/femto molar concentrations, and reduction of the time of analysis [80].

Electrochemical sensors based on graphene screen-printed disposable electrodes were found to be useful for analysis of meat adulteration [84]. For instance, graphene-based electrochemical biosensors combined with a loop-mediated isothermal amplification (LAMP) assay were used for *V. parahaemolyticus toxR* gene detection in seafood products [85]. The nonspecific interaction of the DNA backbone by π-π stacking on graphene-modified screen-printed carbon electrode was used for analysis of amplicons on the picogram level.

#### 3.2.1. Graphene Nanoplatelets (GNPs)

In spite of the superior properties of graphene, its bare form of a monolayer atomic sheet is rarely used for electrochemical sensing applications. The technology of scalable monolayer graphene production based on chemical vapor deposition (CVD) is still in development to become low cost. Moreover, the defects and active sites in graphene are highly desirable for the binding of molecules and increasing sensitivity and selectivity [86]. Thus, bare graphene does not meet these demands because of its ideal crystalline structure, which would require additional treatment and, subsequently, increase the complexity of electrochemical electrode preparation.

**Figure 6:**
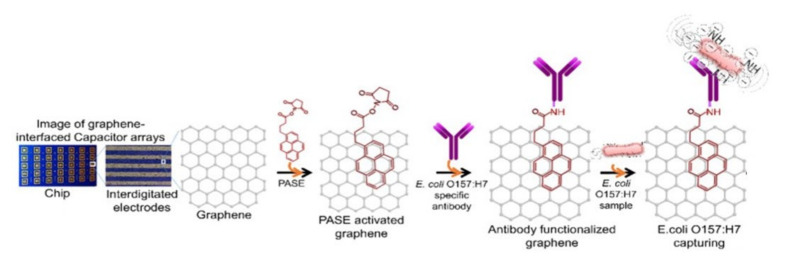
Scheme of a graphene chip and the process of PASE activation and antibody immobilization. Adapted with permission from [87] Copyright 2017, Elsevier.

In contrast, graphene nanoplatelets (GNPs) are a robust graphene-derived material with a 3D structure formed by multiple graphene layers, with properties similar to single-layer graphene. They provide a stable solution in organic solvents without the need for special chemical pre-treatment. GNPs and monolayer graphene were compared in a capacitive sensing platform for foodborne pathogenic *E. coli* O157:H7 detection [87]. The CVD-grown graphene was deposited on a silicon substrate with electrical contacts. Antibodies specific to *E. coli* were immobilized on graphene surface for the selective response during impedance measurements (Figure 6). Higher sensitivity was demonstrated for the monolayer graphene-based sensor, compared to the GNPs, with sensitivities of 10 cells/mL and 100 cells/mL, respectively. Nevertheless, the technology of preparation of less-defective graphene sensing monolayers was more complicated.

GNPs have been applied in the first electrochemical paper-based biosensor. Paper was coated by GNPs and Poly(N-isopropyl acrylamide) (PNIPAm) followed by Au deposition [88]. This biosensor can be applied directly with liquid samples without the use of a bioreceptor. Detection of bacterial cells, Gram negative *E. coli* and positive *S. mutans* and *B. subtilis,* was performed by monitoring the electrical resistance. The achieved detection LoD was only 5 cells/mL.

#### 3.2.2. Graphene Oxide

Graphene derivatives, such as graphene oxide (GO) and reduced graphene oxide (rGO), are preferable materials for electrochemical electrode modification due to a low-cost scalable technology of production and processing in integrated devices [86,89]. The difference between GO and rGO is the number of oxygen molecules present, hence the conductivity. GO shows insulating or semi-conducting behavior, while rGO is electrically conductive but its conductivity also depends on the degree of reduction. Full reduction of GO is still difficult to achieve, while partial reduction of GO is rather easy.

**Table 1:** Detection of pathogens in food with carbon-based integrated electrochemical sensors.

Nanomaterial	Target Pathogen	Working Electrode/Nanomaterial Recognition Complex	EC Technique	Linear Range	LOD	Food Matrix	References
Graphite felt	*E. coli* O157:H7	GF-GCE	OSWV	-	400 cells/mL	Beef	[90]
	*Salmonella*	GF-GCE	OSWV	-	10^3^ cells/mL	-	[91]
Graphene	*E. coli* O157:H7	Cx-Gnfs/ITO	EIS	10^−6^ M–10^−17^ M	1 × 10^−17^ M	-	[92]
	*S. aureus*	ssDNA/GNDs-Zeo/PAD	CV/DPV		0.1 nM	Fruit juice	[93]
	*Vibrio parahaemolyticus*	SPGEs	CV	8 × 10 to 8 × 10^6^ CFU/mL	2 CFU/25 g	Seafood	[85]
	*E. coli* *S. mutans* *B. subtilis*	PNIPAm-GR/Au platform	EIS	10^1^–10^5^ cells/mL	5 cells/mL	Water Milk	[88]
	*E. coli* O157:H7	SiO2-MG SiO2-GNPs	EIS	10–10^7^ cells/mL	10–100 cells/mL	-	[87]
GO	*E. coli* O157:H7	ssDNA/GO/CSGCE	EIS	1 × 10^−14^ to 1 × 10^−8^ M	3.584 × 10^−15^ M	-	[94]
	*Salmonella*	GCE/GO/AuNPs	EIS	2.4–2.4 × 10^3^ CFU/mL	3 CFU/mL	-	[95]
	*S. Typhimurium*	SPCE/rG-GO	EIS	-	10 CFU/mL	Orange juice Water	[96]
rGO	*E. coli* O157:H7	SPCE/PANI-AuNPs-Ab_1_; rGO-NR-Au@Pt-Ab_2_ (measurement of H_2_O_2_ reduction)	CV	8.9 × 10^3^–8.9 × 10^9^ CFU/mL	2840 CFU/mL	Milk Pork	[97]
	*E. coli*	rGO/Al_2_O_3_	FET	1–100 CFU/µL	10^4^ CFU/mL	River water	[98]
	*E. coli*	rGO–CysCu	EIS	10–10^8^ CFU/mL	3.8 CFU/mL	Water Fruit Juice Milk	[99]
	*E. coli* O157:H7	rGO–NR–Au@Pt	CV	4.0 × 10^3^–4.0 × 10^8^ CFU/mL	4.0 × 10^2^ CFU/mL	Pork Milk	[100]
	*L. monocytogenes*	p-rGO/AuNPs/CILE	DPV	1.0 × 10^− 13^–1.0 × 10^− 6^ M	3.17 × 10^− 14^ M	-	[101]
	*Salmonella*	PPy-rGO/GCE/AuNPs	DPV	1.0 × 10^−16^–1.0 × 10^−10^ M 9.6–9.6 × 10^4^ CFU/mL	4.7 × 10^−17^ M DNA 8.07 CFU/mL	-	[102]
	*Salmonella*	rGO-MWCNT	EIS	75 to 7.5 × 10^5^ CFU/mL	25 CFU/mL	Chicken meat	[103]
	*Salmonella*	rGO–CHI	DPV	10–10^6^ CFU/mL	10 CFU/mL	Chicken meat	[104]
	*S. enterica*	rGO-TiO_2_	CV & EIS	10^1^–10^8^ CFU/mL	10 CFU/mL	Chicken meat	[59]
LIG	*S. enterica*	LIG	EIS	25 to 10^5^ CFU/mL	13 ± 7 CFU/mL	Chicken broth	[44]
	*E. coli* O157:H7	AuNPs-LIG	EIS	10^2^−10^8^ CFU/mL	10^2^ CFU/mL	-	[105]
SWCNT	*S. aureus*	SWCNT	EIS	-	10^4^ CFU/mL	-	[106]
	*S. aureus*	SWCNT	DPV	10–10^6^ CFU/mL	13 CFU/mL	Milk	[107]
MWCNT	*Klebsiella pneumoniae Enterococcus faecalis* *E. coli*	ClavA-CNTs-Cys	EIS	10^2^–10^6^ CFU/mL	10^2^ CFU/mL	-	[108]
	*E. coli*	PPy/AuNP/MWCNT/CHI	amperometry	30–30^6^ CFU/mL	30 CFU/mL	-	[109]
	*E. coli* O157:H7	ITO/MWCNT/PEI	EIS	1–10^4^ CFU/mL	1 CFU/mL		[110]
	*S. enterica*	c-MWCNT/AuNP	CV	0.0976–100 ng/µL	0.5 pg/mL	Milk	[111]
	*S. enteritidis*	MWCNT/ITO	CV	10^−1^–10^−8^ CFU/mL	5.5 × 10^1^ CFU/mL 6.7 × 10 CFU/mL	-	[112]
	*S.* Typhimurium	SPCE/MWCNT	DPV	10–10^6^ CFU mL^−1^	7.9 CFU/mL	Milk	[113]
	*S. aureus*	c-MWCNTs-PEI	DPV	-	5 CFU mL^−1^	Milk	[114]
	*Listeria monocytogenes*	MWCNT/fiber electrode	DPV	10^2^–10^5^ CFU/mL	1.07 × 10^2^ CFU/mL	Milk	[115]

Ab, antibody; c-MWCNT, carboxylated multi-walled carbon nanotube; CILE, carbon ionic liquid electrode; CSGCE, chitosan (CS) hybrid nanocomposites modified glassy carbon electrode (GCE); CHI, chitosan; ClavA, antimicrobial peptide clavanin A; CNTs, carbon nanotubes; CV, cyclic voltammetry Cx-Gnfs, carboxylated graphene nanoflakes; DPV, differential pulse voltammetry; EIS, electrochemical impedance spectroscopy; FET, Field-Effect Transistor; GCE, glassy carbon electrode; GF, graphite felt; GND, graphene nano dots; GNP, graphene nanoplatelets; GO, graphene oxide; GR, graphene nanoplatelet; ITO, indium tin oxide; LIG, laser induced graphene; MG, monolayered graphene; MWCNT, multi-walled carbon nanotubes; NR, neutral red; OSWV, Osteryoung square wave voltammetry; p-rGO, partially reduced graphene; PAD, paper analytical device; PANI, regenerative leucoemeraldine base polyaniline; PEI, polyethyleneimine; PNIPAm, poly(N-isopropylacrylamide) polymer; PPy, polyrrole; rG-GO, reduced graphene-graphene oxide; rGO, reduced graphene oxide; rGO-CHI, electrochemically-reduced graphene oxide-chitosan; rGO-CysCu, graphene wrapped copper (II) assisted cysteine hierarchical structure; rGO-TiO_2_, reduced graphene oxide-titanium dioxide; SPCE, screen-printed carbon electrode; SPGE, screen-printed graphene electrodes; SWCNT, single-walled carbon nanotube; Zeo, zeolite.

GO is soluble in aqueous solutions without the need for surfactant addition that is typically the case for graphene and carbon nanotubes. Moreover, the naturally high concentration of defects in GO [116] allow easy functionalization with specific receptor molecules. GO combined with chitosan has been demonstrated to be an excellent means for electron transfer for the detection of short DNA sequences achieving the detection limit of 3.584 × 10^−15^ M [94]. Paper-based sensors with screen-printed electrodes modified by a Nafion/PPy/GO composite were proposed for the detection of lipopolysaccharides (LPSs), which are a marker for Gram-negative bacteria [117]. Raw264.7 macrophage cells were used as a recognition element. The cells were grown in a 3D structure in a Nafion/PPy/GO composite scaffold, serving as a NO gas release to be electrochemically oxidized and detected as a differential pulse voltammetry signal change. GO was shown to be both a good electrical conductor and biocompatible material for cell growth. A sensitivity of 3 pg/mL of LPSs was demonstrated in peach and orange juice.

A rGO-based FET sensor passivated with a layer of Al_2_O_3_ was functionalized with specific antibody immobilized on gold nanoparticles [98]. This sensor was developed to detect *E. coli* in water. Detection was performed by monitoring the change in electrical conductivity of the rGO channel. The LoD was 10^3^ cells/mL. The sensitivity can be improved using rGO modified with cysteine (Cys) in the presence of Cu^2+^ -ions. Such electrochemical immunosensor achieved a LoD of 3.8 CFU/mL of *E. coli* O157:H7 through maintaining the antibody bioactivity [99]. In addition, the biosensor was able to distinguish pathogenic *E. coli* O157:H7 from nonpathogenic *E. coli* strains.

**Figure 7:**
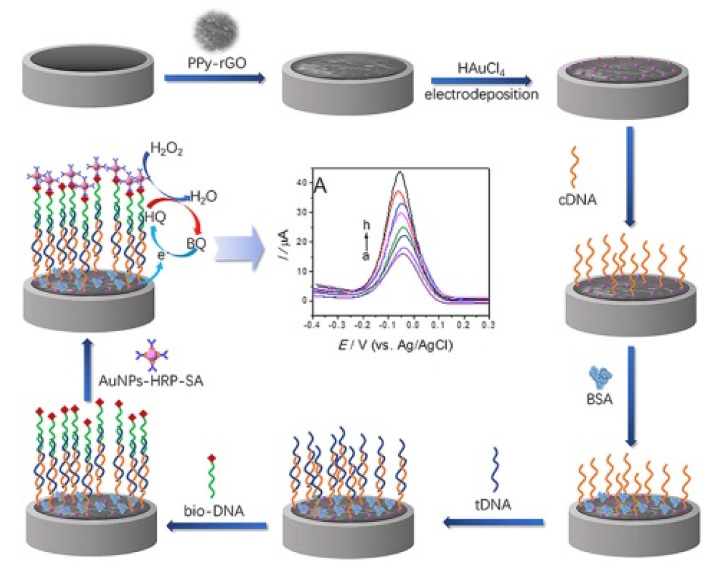
Scheme of the strategy for the electrochemical detection of the *Salmonella inv*A gene. Adapted with permission from [102] Copyright 2019, Elsevier.

A rapid and sensitive electrochemical *inv*A gene biosensor for the detection of *Salmonella* was designed by applying a polypyrrole-rGO nanocomposite on a glassy carbon electrode [102], as shown in Figure 7. Signal amplification was achieved using horseradish peroxidase streptavidin biofunctionalized AuNPs. The LoD was 8.07 CFU/mL with a detection range 9.6–9.6 × 10^4^ CFU/mL.

#### 3.2.3. Laser-Induced Graphene

Recently, novel methods of direct graphene-based electrode writing were applied for portable sensor development [118]. Laser-induced graphene (LIG) is a very simple and scalable technology of porous graphene material production by a local thermal treatment of polymers like polyimide [119]. The obtained material combines the advantages of graphene like a high surface area, electrical conductivity with numerous active centers for surface modifications with different receptors [120]. 

A one step method was proposed to create an electrochemical substrate composed of 3D porous graphene and gold nanoparticles [105]. The aim was to improve the detection performance with a more stable sensor due to the synergic effect of the two nanocomponents. The antibodies were immobilized on the NPs-LIG substrate for the detection of the *E. coli* O157:H7. Despite the use of NPs, the limit of detection achieved was 10^2^ CFU/mL. 

In another study, LIG electrodes modified with polyclonal antibodies were used for the highly selective detection of *Salmonella enterica* serovar Typhimurium [44]. The developed immunoassay demonstrated the linear range of 25 × 10^5^ CFU/mL with a low detection limit of 13 CFU/mL in spiked chicken broth samples and a response time of 22 min. Notably, no special preparation of samples was needed to perform measurements.

#### 3.2.4. Carbon Nanotubes (CNTs)

Carbon nanotubes were used as electrode materials long before the graphene. CNTs are divided into single-walled carbon nanotubes (SWCNTs) and multi-walled carbon nanotubes (MWCNTs) based on the number of graphene sheets [121]. SWCNTs have a diameter range of 0.5 nm to 12 nm but the smallest diameter of SWCNTs is 0.4 nm with different tube lengths starting from several micrometers depending on manufacturing and treatment techniques. MWCNTs consist of multi-rolled layers of graphene inserted one into the other and the number of graphene walls may reach more than 25 walls with a spacing of 0.34 nm. The outside diameter of MWCNTs ranges from 1 nm to 50 nm while the inside diameter is several nanometers. Nevertheless, the problem of good aqueous suspension of carbon nanotubes still prevents their wide usage in integrated electrodes. In addition, proper functionalization of nanotubes is needed which reduces the electrical properties of these nanomaterials.

SWCNT composites were used for highly sensitive detection of bacterial and virus model species *E. coli* O157:H7 and the bacteriophage T7, respectively [122]. The carbon nanotube was used as a transduction element aligned in parallel to bridge two gold electrodes. To provide recognition, SWNTs were functionalized with specific antibodies. The sensor exhibited excellent selectivity, sensitivity and a fast response time of about 5 min in the case of T7 detection, while the response time for the detection of *E. coli* was 60 min.

**Figure 8:**
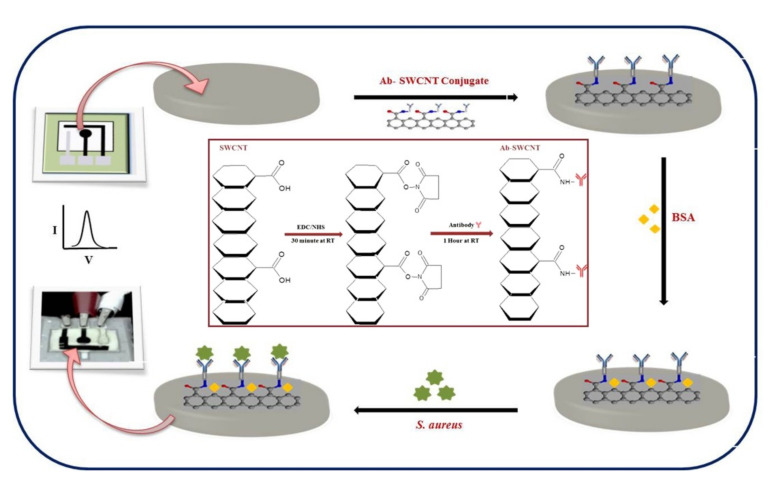
Scheme of the development of an antibody-SWCNT bioconjugate paper-based electrochemical immunosensor. Adapted with permission from [107] Copyright 2017, Elsevier.

SWCNTs with immobilized antibodies were integrated into a disposable bio-nano combinatorial junction sensor for detection of *E. coli* K-12 [123]. Measurements were performed on gold tungsten wires coated with polyethyleneimine with aligned functionalized SWCNTs to form a crossbar junction. Changes in electrical current observed after the SWCNT surface interaction with bacterial cells were monitored to evaluate the sensor’s performance. The biosensor had a LoD of 10^2^ CFU/mL with a detection time of less than 5 min. A low-cost paper-based electrochemical immunosensor was developed utilizing an antibody-SWCNT bioconjugate for rapid detection of *S. aureus* using differential pulse voltammetry (Figure 8), achieving a detection time of 30 min with a detection limit of 13 CFU/mL in spiked milk samples [107].

As a material modification, MWCNTs is better than SWCNTs as it is stiffer, easier and cheaper to produce on a large scale, and several studies have demonstrated to have better sensitivity. Indeed, MWCNTs deposited on an Indium tin oxide (ITO) electrode and modified with aptamers to detect *S. enteritidis* and *S. thyphimuri* achieved a detection limit of 5.5 × 10^1^ CFU/mL and 6.7 × 10^1^ CFU/mL, respectively [112]. Measurements were performed in food samples using cyclic voltammetry and electrochemical impedance spectroscopy techniques to characterize the electrochemical properties and conductivity of the aptasensor. The impedance measured at the aptamer/MWCNT/ITO electrode surface increased after exposure to target *Salmonella* cells, due to the capturing of Salmonella by the immobilized aptamers. A promising electrode substrate was developed with c-MWCNTs to confer an electrical conductivity at bacterial cellulose fibre (BCF) [114]. The BCF was modified with poly- ethyleneimine cation (PEI) to allow the immobilization of phages used as a bioreceptor for *S. aureus*. The LoD of 5 CFU/mL and 2 CFU/mL was found in milk and phosphate buffer saline, respectively, with effective discrimination between dead and live cells and within only 30 min. Moreover, the produced electrodes were maintained stable for over 6 weeks.

Carbon nanomaterials are often used as one of the components in nanocomposite electrochemical biosensors for foodborne pathogen detection. For example, grapheme oxide as part of mesoporous nanocomposite for detection of *E.coli* [46]. A rapid and sensitive detection in the dynamic range from 10^1^ CFU/mL to 10^8^ CFU/mL with a detection limit of 10^1^ CFU/mL of *S. enterica* was achieved with a nanocomposite of rGO and CNT modified with an amino-modified DNA aptamer [124].

The low-cost carbon materials, including graphene and carbon nanofibers, provide a large specific surface area, high electron transfer rate and good catalytic properties, which is of high importance for development of sensing platforms that can be miniaturized for point-of-need testing.

### 3.3. Gold Nanoparticles

**Figure 9:**
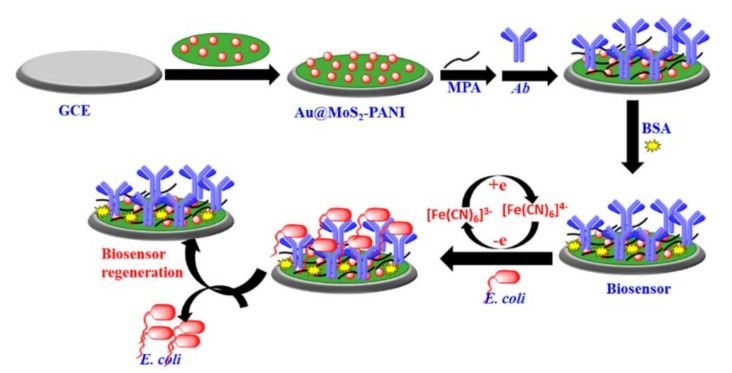
Schematic representation of the label-free electrochemical biosensor based on Au@MoS_2_–PANI. Adapted with permission from [128] Copyright 2021, MDPI.

Gold nanoparticles (AuNPs) have been increasingly used in the design of electrochemical biosensors for their biocompatibility, conductivity, catalytic activity, stability and high surface-to-volume ratio [125]. Deposition of AuNPs onto gold electrodes enables a significant increase in the electrode surface area for target recognition and, consequently, its analytical performance [126,127]. When AuNPs are immobilized on the surface of electrodes made of other materials (such as carbon, graphene, paper, etc.), they increase the surface biocompatibility, promote electron transfer between electrode and immobilized molecules and enable easy bio-conjugation of recognition elements besides increasing the electrode surface area. Raj et al. [128] developed a label-free electrochemical biosensor for the detection of *E. coli* based on a glassy carbon electrode with immobilized a complex of polyaniline nanocomposites (PANI), gold nanoparticles and MoS_2_ (Au@MoS_2_–PANI), in order to increase conductivity, stability and electro-activity of the electrode. The surface of AuNPs were treated with mercaptopropionic acid to covalently immobilize antibodies against *E. coli* and to minimize the nonspecific adsorption on the electrode surface. The biosensor was simple, rapid and specific, with a LoD of 10 CFU/mL and a linear detection range of 10–10^7^ CFU/mL. A schematic representation of this electrode construction is shown in Figure 9. In another study, AuNPs were immobilized on a carbon screen-printed electrode to increase the stability and efficacy of the electrochemical biosensor for the label-free detection of *E. coli* [29]. The modified electrode was treated with N-(γ-Maleimidobutyryloxy) succinimide (GMBS) to create -NHS groups for cross-linking of *E. coli* O157-specific polyclonal antibodies. The analysis showed rapid and efficient pathogen detection with a dynamic range of 10–10^6^ CFU/mL and a LoD of 15 CFU/mL.

The electrocatalytic properties of AuNPs towards hydrogen evolution reaction was employed for rapid and highly sensitive immunodetection of *E. coli* O157:H7 in minced beef and water [129]. The test was performed in a sandwich format where superparamagnetic microbeads modified with the first antibody were used to perform pre-concentration/purification of the bacterial cells from the sample and AuNPs modified with the second antibody provided the catalytic reaction. The method showed a LoD of 457 CFU/mL in minced beef and 309 CFU/mL in water. When compared with a commercial lateral flow kit in terms of LoD, specificity, reproducibility and detection range, the electrochemical method showed clear advantages. Similarly, the magneto-immunoassay and AuNPs as label for electrochemical detection was developed for the detection of *Salmonella enterica* subsp. enterica serovar Typhimurium LT2 (S) in skimmed milk by Alfonso et al. [130]. A magnet is incorporated under the screen-printed carbon electrode to attach magnetic beads carrying *Salmonella* specific antibodies. Beads were added to milk samples to pre-concentrate bacterial cells and then deposited onto the electrode. A sandwich was created using AuNPs modified with antibodies to provide a redox signal. Applying differential pulse voltammetry, a linear range from 10^3^ cells/mL to 10^6^ cells/mL and a LoD of 143 cells/mL was found for skimmed milk samples contaminated with *Salmonella*. AuNP modified screen-printed carbon electrodes were combined with magnetic nanoparticles coupled to specific peptides via a streptavidin interaction to achieve multiplexed electrochemical detection of *Listeria monocytogenes* and *Staphylococcus aureus* with a low detection limit of 9 CFU/mL and 3 CFU/mL, respectively [131].

Magnetic and gold nanoparticles have also been combined in impedance biosensors. For instance, Wang et al. [132] used urease-modified AuNPs to amplify the signal of impedance biosensors implemented with magnetic nanoparticles for the detection of *Listeria monocytogenes.* Bacterial cells captured between magnetic nanoparticles decorated with a monoclonal antibody and AuNPs–urease complex carrying the polyclonal antibody were resuspended in urea to catalyze its hydrolysis into ammonium and carbonate ions. Generated ions were detected by a screen-printed interdigitated electrode. The technique, characterized by low cost and high specificity, gave a linear range from 1.9 × 10^3^ CFU/mL to 1.9 × 10^6^ CFU/mL, and a LoD of 1.6 × 10^3^ CFU/mL, in spiked lettuce samples.

Expensive mono- and poly-clonal antibodies can be replaced with lectins that recognize LPS on the bacterial surface. Oliveira et al. [133] immobilized Cramol L lectin on AuNPs functionalized with l-cysteine. Cramol L is a *Cratylia mollis* lectin used as the recognition interface by making hydrogen bonds with methyl-α-d-mannoside in LPS. To build the biosensor the gold electrode surface was covered by a poly (vinyl chloride-vinyl acetate maleic acid) layer to attach Au-cysteine-Cramol L nanoparticles through the electrostatic interactions. Bovine serum albumin was used to block the remaining non-functionalized electrode surface. The sensor, tested on *E. coli*, *Serratia marcescens*, *Salmonella enterica* and *Klebsiella pneumoniae*, was able to selectively discriminate bacterial species due to their different LPS composition with a high sensitivity.

**Figure 10:**
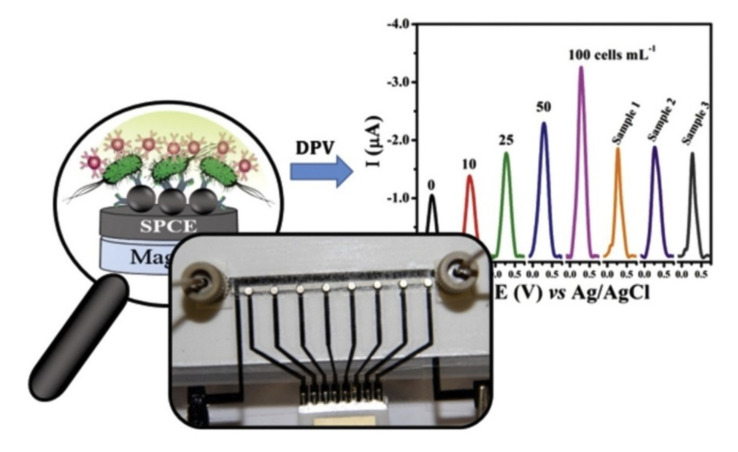
The microfluidic device connectors and the eight magnets externally attached with double-sided tape over each working electrode, together with a schematic representation of detection. Adapted with permission from [134] Copyright 2018, Elsevier.

Although AuNPs based electrochemical biosensors have been extensively employed, their complexity is still an issue limiting the general application, especially in complex food matrices. Usually a multistep procedure, it involves user manual interventions during the test, such as for repetitive washing, loading of samples and reagents. These steps increase the time of analysis and cause imprecise result. Attempts have been made to automatize manual interventions by coupling microfluidic with electrochemical cell. Microfluidic can also enable multiplex detection of different pathogens in the same sample. A disposable microfluidic device for *Salmonella typhimurium* detection in milk was proposed by de Oliveira et al. [134]. The microfluidic device allowed the simultaneous measurement of eight samples by a magneto-immunoassay, as illustrated in Figure 10. The bacteria were captured from the sample by magnetic beads modified with a monoclonal antibody. A sandwich was then completed with AuNPs labeled with a polyclonal antibody. The complex was injected into the device and magnetically placed on the electrode surface. This approach allowed to obtain an easy to use and rapid detection, with a LOD of 7.7 cells/mL.

A sandwich-type electrochemical immunosensor for the detection of *L. monocytogenes* proposed to use 3,4,9,10-perylene tetracarboxylic acid/graphene ribbon nanohybrids as a sensing platform and ferrocene/AuNPs as a signal amplifier [135]. A low detection limit of 6 CFU/mL and linear range of 10–2 × 10^4^ CFU/mL was achieved, showing that incorporation of nanomaterials, such as graphene and AuNPs, enables improved sensing properties.

Electrochemical biosensors based on AuNPs have been successfully applied for virus detection [136]. For instance, the Middle East respiratory syndrome coronavirus (MERS-CoV), which is one of the highly pathogenic viruses, was found to contaminate dairy products [137]. It was detected by the competitive assay carried out on an array of carbon electrodes modified with gold nanoparticles [136]. The electrode array enabled multiplexed detection of different strains of CoVs through the indirect competition between free virus in the sample and immobilized MERS-CoV protein S1 or a fixed concentration of antibody added to the sample. By using ferrocyanide/ferricyanide as a redox probe, voltammetric measurements performed within 20 min showed low detection limit of 1.0 pg/mL for MERS-CoV and high selectivity.

### 3.4. Other 0–3D Nanomaterials

Besides AuNPs, silver nanoparticles (AgNPs) have also been applied for electrochemical detection. For instance, chitosan stabilized AgNPs were applied for electrochemical detection of negatively charged LPS, enabling detection of *E. coli* in the range 10–10^7^ CFU/mL [138].

The high benefits of graphene as a transducer layer for working electrodes in electrochemical biosensors have resulted in an increased interest in the 2D nanomaterial family for application in electrochemical sensing [33]. Semiconductor 2D materials, such as transition metals dichalcogenides (TMDC) and transition metal carbides and carbonitrides (MXenes), have a high surface area and conductivity and possess an intrinsic energy band providing sensitivity to the weak changes in the charge state on electrodes. They can thus greatly improve the performance of electrochemical sensors [139]. A technological process of defect-free 2D materials production is still based on the CVD method, which is an expensive technological process demanding high-purity precursors. In contrast, the richness of defects and boundary grains in MXene production is very simple and inexpensive and better adapted for applications in portable electrochemical devices [140]. Two-dimensional transition metal materials provide a robust sensing surface due to their structural stability and excellent electrochemical properties such as conductivity, catalytic performance and abundant active sites. Previous reviews have discussed the perspective of the electrochemical sensor development based on molybdenum disulfide (MoS_2_) [141] and MXene nanomaterials [142].

**Figure 11:**
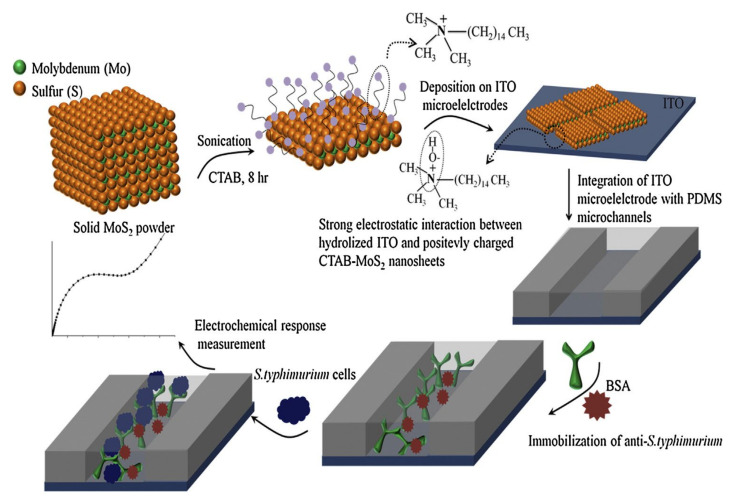
Design of a MoS_2_ based biosensor for *S. typhimurium* detection. Adapted with permission from [145] Copyright 2018, Elsevier.

Hexagonal MoS_2_ is a stratified crystal which has thicknesses equal to the unit cell of the material in a way that each plane of MoS_2_ is made of molybdenum atoms sandwiched between sulfur atoms and stabilized by van der Waals bonds. Two-dimensional MoS_2_ is obtained when the material is exfoliated into one or a limited number of layers. Two-dimensional MoS_2_ shows remarkable electronic, optical, mechanical and chemical characteristics that also make it advantageous for biosensing applications. In addition, when grown into planes with relatively large lateral dimensions, 2D MoS_2_ is particularly stable in liquid and oxygen containing gaseous media which facilitate their utilization when incorporated into biosensing structures [143]. An electrochemical lab-on-paper genosensor was developed based on carbon ink screen-printed on cellulose paper with a working electrode modified by drop-casted MoS_2_ nanosheets for detecting *Salmonella*-specific DNA [144]. The sensitivity of the MoS_2_-modified electrode was increased more than 10 times due to the enhanced transfer rate of charge carries and unique electron transfer kinetics in MoS_2_. To provide the selectivity towards *Salmonella*-DNA, the specific complementary DNA probe was immobilized on MoS_2_ for on electrode hybridization. The LoD of 20 nM was obtained. Another method for foodborne pathogen detection was suggested based on antibody immobilized onto a microfluidic chip. Exfoliated MoS_2_ nanosheets in the presence of a surfactant were deposited on ITO electrodes integrated with a microfluidic channel to develop an impedimetric biosensor [145], as shown in Figure 11. The specific antibody directed against *Salmonella typhimurium* was immobilized on a MoS_2_/ITO electrode treated with glutaraldehyde. Proper functionalization of 2D nanosheets and optimization of the procedure for antibody molecules association with MoS_2_ yielded superior electron conduction and resulted in a 1.5 CFU/mL limit of detection.

Metal organic frameworks (MOFs) are a rapidly emerging new class of microporous materials with a wide range of promising applications [146,147]. They basically represent 2D or 3D porous materials assembled using metal cation salts or clusters bridged with polydentate organic ligands with coordination type connections, though 0D and 1D nanostructures are being synthesized also [148]. They have a very high surface area, high pore volume, high porosity and surface functionality and an easily tunable structure. Nanoscale MOFs combine the properties of both MOFs and nanostructures. MOFs are often used to design complex nanocomposite materials through a controlled assembly of MOF nanoparticles, such as NP@MOFs. Two-dimensional metal organic frameworks (MOF) have recently come into focus for biosensing applications [149]. The possibility of tuning their properties in a controllable way and the extremely high surface area is expected to outperform traditional electrochemical sensors. Still, their poor conductivity demands a combination with other highly conductive nanomaterials in the form of nanocomposites. Sensitive impedimetric detection of *E. coli* in the range 2.1 × 10^2^–2.1 × 10^8^ CFU/mL with a detection limit of 4 CFU/mL was achieved by combining a (MOF) with a conducting polymer (CP) and PEDOT on modified carbon screen-printed electrodes [150]. Copper (Cu)-MOFs were directly self-assembled and deposited onto a glassy carbon electrode, followed by in situ reduction of AuNPs on the MOF surface and conjugated with a DNA aptamer enabling detection of *S. aureus* in the range 7–7 × 10^6^ CFU/mL [151]. Graphene and a zirconium based MOF (UiO-67) were combined together with an aptamer loaded AuNP-horseradish conjugate to detect *S. typhimurium* in spiked milk samples with a detection limit of 5 CFU/mL [152].

Quantum dots (QDs), carbon dots (CDs) and graphene quantum dots (GQDs) are another category of nanomaterials with great potential for application in electrochemical biosensing of foodborne pathogens [27,31]. QDs have been used to modify the structure of polymeric nanodendrons for direct culture-free electrochemical detection of *Salmonella* in milk with a detection limit of 4 CFU/mL [153]. GQDs combine characteristics both from graphene and carbon dots, offering great versatility for modification with other modifiers and nanomaterials besides low cost, low toxicity, high solubility and good electronic properties. Photoelectrochemical sensing represents an integration of electrochemistry and photochemistry offering high sensitivity, robustness, low cost and simple instrumentation. For instance, GQDs doped with nitrogen were coupled with non-metallic two dimensional hydrated defected tungsten oxide to design a photoelectrochemical aptamer biosensor for *E.coli* detection in the range 0.1–10^4^ CFU/mL with a low detection limit of 0.05 CFU/mL [154].

## 4. Conclusion and Perspectives

The present review summarizes the unique properties of 0–3D nanomaterials and their application in the design of electrochemical biosensors for foodborne pathogen detection. Despite the progress and advances in culture based and molecular methods for foodborne pathogen detection, challenges remain for their practical application because they still do not reach the sensitivity, fast response time and low cost needed. Effective foodborne pathogen monitoring that will enable efficient risk assessment and outbreak prediction has to be rapid, ultrasensitive, specific and affordable to be applied in low-resource settings. Electrochemical biosensors offer an exciting opportunity to realize immediate and continuous pathogen detection for on-site risk evaluation. We have highlighted examples showing that electrochemical methods can release results within several hours or even several minutes. Besides, a wide variety of strategies used to improve sensitivity are presented. Some foodborne pathogens, such as *Campylobacter*, *E. coli* O157 and *L. monocytogenes,* have very low infectious doses of a dozen to several hundred cells. Consequently, it is of high importance that low contaminated food items can be identified. Amplification of the detection signal using nanomaterials as electronic conductors, carriers or catalysts enable electrochemical biosensors to exhibit LoDs as low as a single colony forming unit (CFU/mL) or several femto M or even atto M ranges and linear ranges of several orders of magnitude. Although the presented nanomaterials can be used with other detection techniques, such as plasmonic and fluorescent, electrochemical biosensors have the advantage of simple utilization by persons without previous training, versatile detection schema providing a wide range of applications and easy miniaturization [155,156,157]. Moreover, the inexpensive and miniaturized electrochemical devices in handheld formats are excellent candidates for on-site application.

Cyclic voltammetry, differential pulse voltammetry, square wave voltammetry and electrochemical impedance spectroscopy are the methods mostly used for detection of pathogens and their toxins in food samples. At the same time, nanomaterials are generally applied with these methods to enhance the detection signal. Planar gold electrodes are the most commonly used working electrodes. However, nanomaterials with their outstanding properties that arise from their small dimensions and surface reactivity are applied to alleviate the limitations of electrochemical biosensors, such as slow recognition time, low biocompatibility or instability. In addition, some nanomaterials, such as gold nanoparticles or graphene, may tune the electrode properties and offer a variety of surface engineering strategies and functionalization to attach biological entities assuring recognition (e.g., antibodies, aptamers, ssDNA, phages). One of the trends in recent electrochemical biosensors for pathogen detection is to combine several nanomaterials as nanocomposites in electrode design to obtain remarkable synergic effects leading to improved sensing performances. Moreover, specific nanomaterials, such as graphene or metal oxide nanoparticles, have inherent antibacterial activity. Biosensors integrating such nanomaterials are multi-functional, providing not only pathogen identification and quantification but also their elimination.

During the past decade, significant progress has been made in the biosensors field to advance electrochemical devices, taking into account food industry demands. Hence, further improvement is needed to facilitate wide practical applicability of biosensors for detecting foodborne pathogens. Biocompatibility is one high concern. The electrode design, surface modification and functionalization integrating nanomaterials are of great promise to improve the stability and compatibility of electrodes in biological environments for an extended period. Besides, due to the diversity of foodborne pathogens and the possibility of food co-contamination by various microorganisms, multi-electrode detection devices that exhibit different biorecognition elements for simultaneous multiplex detection without mutual interference are also needed for improved detection efficiency. In addition, most electrochemical biochips are designed only for single utilization. We expect that future studies will anticipate environmental friendliness and resource conservation and will include reusability of electrodes in sensor design.

Analytical performances of the majority of presented biosensors refer only to LoD and linear range of detection. However, other parameters, notably accuracy, repeatability, precision, and specificity, should be also investigated and improved to enable sensors general accessibility. Furthermore, the synthesis procedure of integrated nanomaterials has to be optimized to obtain rigorous protocols for mass production and strict quality control of the material without chemical impurities that can alter sensing properties or induce environmental pollution.

Finally, pathogen detection in foods requires sample treatment and transport to the electrode surface, which may cause analyte loss. For this reason, advanced electrochemical biosensors based on nanomaterials that detect pathogens and their toxins in complex matrices without important interferences should be designed. Coupling detection with a microfluidic system for sample handling holds great potential.

In the future, additional excellent nanomaterial-based electrochemical biosensors will emerge, and new design and solutions will be proposed. It is evident that the field of nanomaterials is making tremendous progress and significantly affects biosensor development. Active collaboration between material scientists, microbiologists, electrochemists and device developers in the fields of nanotechnology and food science will result in point-of-need diagnostic devices integrating electrochemical biosensors, microfluidics and nanomaterials. Such devices will offer the food industry food safety analyses and foodstuff screening that can be performed during all phases, from production, packaging, storing and distribution to consumption.

## Data Availability

This study did not report any data.
